# Anal High-risk HPV and Liquid-based Cytology of Immunocompetent Brazilian Women with Genital High-risk HPV

**DOI:** 10.1055/s-0042-1742405

**Published:** 2022-02-09

**Authors:** Karla Maria Rêgo Leopoldo Melo, José Eleutério Junior, Raquel Autran Coelho Peixoto, Karinne Cisne Fernandes Rebouças, Renata Mirian Nunes Eleutério

**Affiliations:** 1 Faculdade de Medicina, Departamento de Saúde Materno-Infantil, Universidade Federal do Ceará, Fortaleza, CE, Brazil; 2 Faculdade de Biomedicina, Departamento de Biomedicina, Unichristus, Fortaleza, CE, Brazil

**Keywords:** cytology, papillomaviridae, colposcopy, anus neoplasm, polymerase chain reaction, citologia, papilomaviridae, colposcopia, neoplasias do ânus, reação em cadeia da polimerase

## Abstract

**Objective** The purpose of this study was to compare the frequency of the occurrence of high-risk human papillomavirus (HPV) and abnormal anal cytology in immunocompetent women with and without HPV-induced genital lesions.

**Methods** This analytical cross-sectional, observational study was conducted between July 2017 and December 2018 in a specialized outpatient clinic of a tertiary hospital in Fortaleza, CE. Fifty-seven immunocompetent women with and without genital intraepithelial lesions were assessed; they were divided into two groups: group 1 was comprised of women with HPV-associated genital lesions ( *n* = 26), and group 2 was comprised of those without HPV-associated genital lesions ( *n* = 31). Samples for liquid-based cytology and high-risk DNA-HPV polymerase chain reaction real-time tests were collected from the cervix and anus. All cases were evaluated using high-resolution anoscopy; biopsies were performed when required. The Fisher exact and chi-squared tests were applied for consolidated data in the contingency table, and the Student *t* -test and Mann-Whitney U-test for independent variables.

**Results** Anal high-risk HPV infections were more frequent in group 1 (odds ratio [OR], 4.95; 95% confidence interval [CI], 1.34–18.3; *p* = 0.012), along with concomitant high-risk HPV infections in the uterine cervix and the anus (OR 18.8; 95% CI, 2.20–160; *p* < 0.001). The incidence of high-risk cervical HPV infection was associated with high-risk anal HPV infection (OR, 4.95; 95% CI, 1.34–18.3; *p* = 0.012). There was no statistical difference concerning abnormal anal cytology or anoscopy between the groups, and no anal intraepithelial lesion was found in either group.

**Conclusion** Immunocompetent women with HPV-associated genital lesions and high-risk cervical HPV were more likely to have high-risk anal HPV.

## Resumo

**Objetivo** O objetivo deste estudo foi comparar a frequência de papilomavírus humano (HPV) de alto risco e citologia anal anormal em mulheres imunocompetentes com e sem lesões genitais induzidas por HPV.

**Métodos** Este estudo transversal analítico e observacional foi realizado entre julho de 2017 e dezembro de 2018 em um ambulatório especializado de um hospital terciário em Fortaleza, CE. Cinquenta e sete mulheres imunocompetentes com e sem lesões intraepiteliais genitais foram avaliadas. Foram divididas em dois grupos: grupo 1, composto por mulheres com lesões genitais associadas ao HPV ( *n* = 26) e grupo 2, composto por mulheres sem lesões genitais associadas ao HPV ( *n* = 31). Amostras para citologia em meio líquido e testes de reação em cadeia da polimerase em tempo real para DNA-HPV de alto risco foram coletadas do colo do útero e do ânus. Todos os casos foram avaliados por anuscopia de alta resolução; sendo realizada biópsia quando necessária. Os testes exatos de Fisher e qui-quadrado foram aplicados para dados consolidados na tabela de contingência; o teste t de Student e o teste U de Mann-Whitney foram aplicados para variáveis independentes.

**Resultados** As infecções anais por HPV de alto risco foram mais frequentes no grupo 1 (razão de chances [RC], 4,95; intervalo de confiança [IC] de 95%, 1,34–18,3; *p* = 0,012), assim como infecções concomitantes por HPV de alto risco em colo uterino e ânus (RC 18,8; IC de 95%, 2,20–160; *p* < 0,001). A incidência de infecção de HPV cervical de alto risco foi associada à infecção de HPV anal de alto risco (RC, 4,95; IC de 95%, 1,34–18,3; *p* = 0,012). Não houve diferença estatística em relação à citologia anal anormal ou anuscopia entre os grupos, e não houve caso de lesão intraepitelial anal em nenhum dos grupos.

**Conclusão** Mulheres imunocompetentes com lesões genitais associadas ao HPV e com HPV cervical de alto risco foram mais propensas a ter HPV anal de alto risco.

## Introduction

The incidence of anal cancer has increased in the last few decades, especially in women; the occurrence of anal cancer is currently more frequent in women than in men. 1 2 As in cervical cancer, a *human papillomavirus* (HPV) infection may be crucial in the development of anal cancer. 3 4 High-risk oncogenic HPV (Hr-HPV) is present in 99% of cervical cancers and 90% of anal cancers. The HPV subtypes 16 and 18 are associated with ∼ 70% of cervical cancers and 78% of anal cancers. 4

A cervical HPV infection increases the risk of anal infection of the same viral type. 5 6 7 8 A persistent Hr-HPV infection increases the risk of an anal high-grade squamous intraepithelial lesion (HSIL) and anal cancer. 7 8 An anal HSIL is considered a precursor of anal cancer. 9

Considering that screening programs for cervical cancer with Papanicolaou smears have been associated with a decrease in the incidence and mortality rates of this cancer, anal cytology has been used as a screening method for anal HSILs in high-risk individuals. Patients with abnormal anal cytology undergo a high-resolution anoscopy (HRA) or colposcopic evaluation of the anus and biopsy when required. 10 Women with a history of a genital squamous intraepithelial lesion (SIL) are at increased risk for anal HSILs and cancer as compared with the general population, and some authors recommend anal cytological screening for these women. 10 11 12 13

In Brazilian women, there are few studies on the prevalence of anal Hr-HPV and anal SILs. The purpose of this study was to evaluate the frequency of Hr-HPV infection and abnormal anal liquid-based cytology among immunocompetent women with and without a genital SIL.

## Methods

This quantitative, observational, analytical cross-sectional study was conducted at Maternidade Escola Assis Chateaubriand (a tertiary hospital in Fortaleza, CE, Brazil) from July 2017 to December 2018. Fifty-seven women without immunosuppression over 18 years of age were assessed. The women were divided into two groups: group 1 ( *n* = 26) had a histopathological diagnosis of genital HSILs or low-grade SILs (LSIL), and group 2 ( *n* = 31) had no genital SIL. Pregnant women, those with condylomas or genital cancer, or those undergoing radiotherapy or chemotherapy were excluded from the study.

After obtaining written informed consent, each patient answered a standardized questionnaire that enquired about age, parity, sex, history of anal intercourse, total number of sexual partners, history of sexually transmitted diseases (STDs), cervical cytologic abnormalities, vulvar warts, smoking status, and contraception.

The participants underwent a gynecological examination performed by one of the three researchers, and samples were collected from the cervix using a detachable brush, the Rovers Cervex-Brush Combi (THERAPAK. Claremont, CA, USA). The brush head was detached and placed in a vial that contained a SurePath (Becton Dickinson and Company, Franklin Lakes, NJ, USA) preservative liquid. The buttocks of the participants were separated to expose the anus, and a cytobrush was inserted 4 cm into the anal canal. It was removed with care to avoid touching the perianal region and placed in a vial containing a SurePath preservative liquid. Both samples were sent for cervical and anal cytology and high-risk DNA HPV tests. All participants underwent cervical colposcopy and high-resolution anoscopy (HRA). Biopsies were performed when necessary.

Cervical and anal specimens were sent to a reference laboratory in duly labeled tubes. An aliquot of the harvested material was processed according to the manufacturer's recommendations for SurePath slides. After staining and mounting, the slides were examined by an experienced cytopathologist (J. E. J.). The cytological diagnoses were performed according to the Bethesda 2001 system. 14 The polymerase chain reaction (PCR) real-time tests for high-risk oncogenic types of HPV were performed according to the manufacturer's specifications (CEPHEID XPERTTM method - CEPHEID, Sunnyvale, CA 94089. USA) in cervical and anal specimens, including probes for detection of HPV 16, HPV 18/45, and 11 other types of high-risk HPV (types 31, 33, 35, 39, 51, 52, 56, 58, 59, 66, and 68).

After the collection of the cervical and anal samples, all the patients underwent an anogenital examination that included a colposcopy and an HRA using 5% acetic acid solution. Biopsies were performed for abnormal findings using the Medina biopsy forceps (3–5 mm tip) (RHOSSE. Ribeirão Preto, SP, BRAZIL); the biopsied fragments were fixed in a 10% formaldehyde solution in separate containers according to the biopsy site and were labeled and sent to the pathology laboratory of Universidade Federal do Ceará.

An Excel spreadsheet (Microsoft Corp. Redmond, WA, USA) was organized to study the variables, and the data were exported to the Jamovi 0.9.5.12 program, where the Pearson chi-squared and Fisher exact tests were applied to the consolidated data in the contingency table, as well as the Student t and Mann-Whitney tests for numeric variables. Statistical significance was set at *p* < 0.05.

This study was approved by the ethics research committee of the institution (44961515.5.0000.5050) and was funded by the Conselho Nacional de Desenvolvimento Científico e Tecnológico, Ministério da Ciência, Tecnologia e Inovação of Brazil.

## Results

Of the 57 women studied, 26 presented with genital SILs, 24 with cervical SILs, and 2 with vaginal SILs (group 1); 31 had no genital SIL (group 2). The sociodemographic features (age and number of pregnancies), behavioral variables (smoking status, number of sexual partners, sexual practices, and a sexually transmitted disease [STD] history with the exception of sexarche), and anal symptoms were similar for both groups of women with and without SILs ( Table 1 ).

**Table 1:** Characteristics of women with genital squamous intraepithelial lesions (group 1) and women without them (group 2)

	GROUP 1 (N:26)	GROUP 2 (N:31)	P
Age (mean [SD])	32.7 (9.27)	35.3 (8.80)	0.276*
Sexarche (median [p25; p75])	16 (14; 18)	17 (16; 20)	0.045**
Sexual partners (median [p25; p75])	2 (1; 3.25)	2 (2; 4)	0.731**
Pregnancies (median [p25; p75])	1.5 (0; 3.75)	2 (0.5; 3)	0.915**
Anal intercourse (n [%])	12/22 (54.5%)	17/29 (58.6%)	0.770***
Smoking status (n [%])	3/25 (12%)	2/30 (6.7%)	0.650****
History of STD (n [%])	9/24 (37.5)	9/30 (30%)	0.560***
Anal disease (n [%])	6/22 (27.3%)	9/29 (31%)	0.770***
Anal bleeding (n [%])	6/22 (27.3%)	11/29 (37.9%)	0.420***
Bisexual partner (n [%])	1/22 (4.5%)	1/29 (3.4%)	> 0.999****

Abbreviations: SD, standard deviation; SIL, squamous intraepithelial lesion; STD, sexually transmitted disease.

* Student t-test; ** Mann-Whitney U-test; *** Pearson chi-squared test, **** Fisher exact test.

Table 2 shows the frequency of high-risk oncogenic HPV types, according to the site of sample collection, in the studied groups.

**Table 2:** Distribution of high-risk oncogenic HPV type among women with genital squamous intraepithelial lesion (group 1) and women without genital SIL (group 2)

	GROUP 1		GROUP 2	
	Cervix	Anus	Cervix	Anus
High-risk oncogenic HPV	76.9%	42.3%	19.4%	12.9%
HR HPV*	65.4%	38.5%	12.9%	9.7%
HPV 16	15.4%	15.4%	3.2%	6.5%
HPV 18	3.8%	0%	6.5%	3.2%

Abbreviations: HPV, human papillomavirus; SIL, squamous intraepithelial lesion.

* HR HPV: types of Hr-HPV 31,33, 35, 39, 51, 52, 56, 58, 59, 66 e 68.

Cervical and anal high-risk oncogenic HPV infection was more frequent in women with genital SILs ( Table 3 ).

**Table 3:** High-risk oncogenic human papillomavirus infection in the cervix and anus in women with genital squamous intraepithelial lesions (group 1) and in women without them (group 2)

	High-risk oncogenic HPV n (%)		Total	Odds ratio (OR)	95% CI	*p* -value*
	GROUP 1 (n:26)	GROUP 2 (n:31)				
Uterine cervix	20 (76.9%)	6 (19.4%)	26 (45.6%)	13.9	3.88–49.7	< 0.001
Anus	11 (42.3%)	4 (12.9%)	15 (26.3%)	4.95	1.34–18.3	0.012

Abbreviation: HPV, human papillomavirus.

* Pearson chi-square.

Despite the association of genital SILs with anal high-risk oncogenic HPV, there was no association of genital SILs with abnormal anal cytology: 19.2% of women in group 1 and 9.7% of women in group 2 had abnormal anal cytology ( *p* = 0.45). There was also no association between genital SILs and abnormal anoscopy: 21.7% in group 1 and 20.7% in group 2 had an abnormal anoscopy ( *p* = 0.95). Women with an abnormal anoscopy underwent anal biopsy; there was no histopathological examination of anal SILs in either group.

Women with genital SILs (group 1) were more likely to test positive for concomitant cervical and anal high-risk oncogenic HPV ( *n* = 10 [38.5%]) than women without genital SILs (group 2) ( *n* = 1 [1.32%]) ( *p* < 0.001; OR 18.8, CI 95% [2.20–160]); however, both cervical and anal oncogenic HPV positivity was not associated with abnormal anal cytology or abnormal anoscopies ( *p* = 0.17, *p* = 1.00, respectively).

Regardless of the study group, high-risk cervical HPV infection was associated with high-risk anal HPV infection. This association did not occur with the types of HPV 16 and HPV 18/45 when evaluated alone ( Table 4 ).

**Table 4:** Association between high-risk oncogenic human papillomavirus positivity in the anus and in the cervix of study participants

ANUS HPV GENOTYPE	Similar HPV in cervix		Odds ratio (OR)	95% CI	*p* -value
	Positive	Negative			
HPV 16, 18/45 or HPV HR*	42.3% (11/26)	12.9% (4/31)	4.95	1.34–18.3	0.012**
HR HPV*	47.6% (10/21)	8.3% (3/36)	10	2.32–43	0.002***
HPV 16	40% (⅖)	7.7% (4/52)	7.8	0.99–61.5	0.084***
HPV 18/45	33.3% (⅓)	0% (0/54)	65.4	2.09–2045	0.053***

Abbreviation: HPV, human papillomavirus.

* HR HPV: types of HPV 31,33, 35, 39, 51, 52, 56, 58, 59, 66 e 68.

** Pearson chi-squared test. *** Fisher exact test.

Anal sexual intercourse was not associated with anal oncogenic HPV infection ( *p* = 0.7), abnormal anal cytology ( *p* = 1.0), or abnormal HRA ( *p* = 0.7).

Anal high-risk oncogenic HPV infection was not associated with abnormal anal cytology ( *p* = 0.19) or abnormal HRA ( *p* = 0.71).

Women with HSIL cervical cytology were more likely to have an abnormal anal cytology than women with normal, atypical squamous cells of undetermined significance (ASC-US), atypical squamous cells cannot exclude HSIL (ASC-H), or LSIL cervical cytology ( *p* = 0.005; OR 15.3, CI 95% [2.50–93.9]).

## Discussion

A growing number of studies have shown that HPV-induced genital lesions from surrounding sites increase the risk of anal SILs and anal carcinoma. 5 10 Further research is warranted on the incidence of anal SILs and anal cancer in women with genital SILs, including populations other than that considered in this study. This will help define the group in which screening for anal lesions is essential, considering the cost-effectiveness of the test.

This study showed that women with genital SILs had more anal oncogenic HPV than women without genital SILs (42.3% versus 12.9%). A systematic review in 2015 found that the frequency of anal HPV infection in HIV-negative women with genital SILs ranged from 23 to 86%. 15 A higher frequency of anal oncogenic HPV infection in women with genital SILs compared with women without SILs was also observed in other similar studies. 5 16 17 The positivity of anal HPV seems to vary considerably according to the group studied, and most studies on the screening of anal intraepithelial lesions have been performed with HIV-positive and men who have sex with men (MSM). Studies have shown that women with genital SILs have a higher risk of anal SILs and anal carcinoma. 10 18 19

Similar to a previous study, the frequency of concurrent cervical and anal oncogenic HPV infection was higher in women with genital SILs compared with women without SILs. 17 Despite showing concomitant oncogenic HPV in the cervix and anus of 4.4% of women with a history of genital SILs and in none of the women without this history, a previous study showed no statistical difference in the incidence of oncogenic HPV in women with and without a history of genital SIL. 16 The higher positivity of concomitant infection in our study compared with this previous study was probably because women in our study had a genital SIL at the time of sample collection, and not just a prior history of SILs.

Another finding in the study was that cervical oncogenic HPV infection was associated with an anal oncogenic HPV infection. This association may be explained by the multicentricity of the HPV infection, which, once present at a genital site, may also affect the anus. A recent systematic review of 36 studies also confirmed the association of anal oncogenic HPV infection in women with cervical oncogenic HPV infection, also seen when HPV 16 infections were studied alone, in both HIV-positive and HIV-negative women. 20

In this study, the sociodemographic characteristics, and the clinical data of the two groups were not significantly different, which may have decreased the chance of bias. In other studies comparing groups of women with and without genital SILs, there were differences in the smoking status, number of sexual partners, and history of anal intercourse in these groups, with anal intercourse being more frequent in women with genital SILs. 5 16 17 18 These factors alone may increase the presence of anal Hr-HPV, abnormal anal cytology, and anal SILs. 3 4 10 19

The occurrence of abnormal anal cytology was more frequent in women with genital SILs than in those without SILs; however, this difference may not be significant because all women included in the genital SIL group had an ongoing histopathological diagnosis of a genital SIL, not just a past medical history of genital SILs, which limited the sample. Previous studies that showed a difference in abnormal anal cytology in women with or without SIL included women who only had a history of previous genital SIL. 5 16

The association between HSIL cervical cytology and abnormal anal cytology was also found in previous studies with both HIV-positive and HIV-negative women. 20 21

Regarding HRA, there were few abnormal results in both the groups, unlike previous studies. 11 22 However, anoscopy is a relatively tricky examination with a strict learning curve; thus, the number of abnormal results in our research may have been underestimated. 23

In our study, no anal SILs were found on histopathological examination in either group, and the higher frequency of anal oncogenic HPV infection without anal SIL may have been because it takes a longer time for anal SILs to develop from a primary HPV infection. In other studies that assessed anal HSILs in women with genital SILs, the prevalence of anal HSILs in patients with genital SILs ranged from 3.2 to 8.3%. However, in these studies, the population sample was higher. 5 11 22 24 25 26 27 In addition, some of these studies included women with genital cancer and/or women who tested positive for HIV, unlike the present study, which evaluated women with genital LSILs and HSILs and those without SILs; this may have caused the higher prevalence of anal HSILs in patients with genital SILs in these studies. 11 22 27 The absence of anal SILs is associated with anal HPV infections caused by types of HPV other than HPV 16 and HPV 18, which are more aggressive than other types of HPV. A previous study that assessed 166 HIV-negative women with genital SILs found no anal HSILs on histopathological examination; in this study, the frequency of anal oncogenic HPV infection was 64.5%; HPV types other than HPV 16 and HPV 18 were detected in 50.5% of cases. 28

Similar to previous studies, no association was found between anal intercourse and anal high-risk oncogenic HPV infection, abnormal anal cytology, and HRA in this study. 19 28 However, other researchers have demonstrated an association between anal sexual intercourse and anal oncogenic HPV infection, abnormal anal cytology, and anal HPV persistence. 10 21 A national, cross-sectional study involving immunocompetent women with HPV-induced genital lesions also found an association between anal intercourse and the presence of anal SILs. 25 It is possible that the size of the studied population in the present study caused the absence of association between anal intercourse and anal high-risk oncogenic HPV infection, abnormal anal cytology, and HRA.

Some limitations must be considered when interpreting the data. The accuracy of the test results could be influenced by the quality of the samples. In addition, limited association analyses can be performed in the cross-sectional study design. However, our results provide important evidence regarding the prevalence of anal Hr-HPV infection and anal SILs among immunocompetent Brazilian women.

It is important to understand whether screening for anal HSIL and treatment of this precursor condition prevents anal cancer. Studies are currently underway to address this issue. Although some studies suggest a higher risk of anal intraepithelial lesions in women with genital SILs, the efficacy of screening and treatment of anal cancer precursors in preventing the onset of cancer has not been established. This is an essential topic for future long-term studies. However, until better evidence can be collected, many experts choose to screen and treat individuals at risk for anal cancer, which requires extensive training in high-resolution anoscopy. 29

## Conclusion

Our study demonstrated that anal oncogenic HPV infection occurred more frequently in immunocompetent women with genital SILs than in women without genital SILs. Women with genital SILs tended to have a higher frequency of occurrence of abnormal anal cytology; however, the difference in occurrence of an abnormal anal cytology in women with and without a genital SIL was not statistically significant. Cervical oncogenic HPV infection was associated with anal oncogenic HPV infection, and the results of cervical cytology of HSIL were associated with an abnormal anal cytology. No anal SILs were found on histopathological examination. Therefore, it was impossible to correlate anal SILs with anal cytology or high-risk of anal HPV infection. Further studies are necessary to confirm this association and to determine whether screening and treatment of these lesions in this population of women with genital SILs can prevent anal cancer.
